# Accelerating the Discovery of Anticancer Peptides through Deep Forest Architecture with Deep Graphical Representation

**DOI:** 10.3390/ijms24054328

**Published:** 2023-02-21

**Authors:** Lantian Yao, Wenshuo Li, Yuntian Zhang, Junyang Deng, Yuxuan Pang, Yixian Huang, Chia-Ru Chung, Jinhan Yu, Ying-Chih Chiang, Tzong-Yi Lee

**Affiliations:** 1 Kobilka Institute of Innovative Drug Discovery, School of Medicine, The Chinese University of Hong Kong (Shenzhen), 2001 Longxiang Road, Shenzhen 518172, China; 2 School of Science and Engineering, The Chinese University of Hong Kong (Shenzhen), 2001 Longxiang Road, Shenzhen 518172, China; 3 Warshel Institute for Computational Biology, School of Medicine, The Chinese University of Hong Kong (Shenzhen), 2001 Longxiang Road, Shenzhen 518172, China; 4 School of Medicine, The Chinese University of Hong Kong (Shenzhen), 2001 Longxiang Road, Shenzhen 518172, China

**Keywords:** anticancer peptide, drug discovery, feature extraction, deep forest, sequence analysis

## Abstract

Cancer is one of the leading diseases threatening human life and health worldwide. Peptide-based therapies have attracted much attention in recent years. Therefore, the precise prediction of anticancer peptides (ACPs) is crucial for discovering and designing novel cancer treatments. In this study, we proposed a novel machine learning framework (GRDF) that incorporates deep graphical representation and deep forest architecture for identifying ACPs. Specifically, GRDF extracts graphical features based on the physicochemical properties of peptides and integrates their evolutionary information along with binary profiles for constructing models. Moreover, we employ the deep forest algorithm, which adopts a layer-by-layer cascade architecture similar to deep neural networks, enabling excellent performance on small datasets but without complicated tuning of hyperparameters. The experiment shows GRDF exhibits state-of-the-art performance on two elaborate datasets (Set 1 and Set 2), achieving 77.12% accuracy and 77.54% F1-score on Set 1, as well as 94.10% accuracy and 94.15% F1-score on Set 2, exceeding existing ACP prediction methods. Our models exhibit greater robustness than the baseline algorithms commonly used for other sequence analysis tasks. In addition, GRDF is well-interpretable, enabling researchers to better understand the features of peptide sequences. The promising results demonstrate that GRDF is remarkably effective in identifying ACPs. Therefore, the framework presented in this study could assist researchers in facilitating the discovery of anticancer peptides and contribute to developing novel cancer treatments.

## 1. Introduction

Cancer is one of the most severe diseases in the world. Although significant progress has been made in cancer treatment, there are no specific drugs for most cancers [1]. Conventional cancer treatments, such as radiotherapy and chemotherapy, cannot target cancer cells directly, resulting in severe side effects [2]. Therefore, there is a growing demand for developing effective cancer treatments [3].

Peptide-based therapy has gained much attention for its high specificity and low risk of inducing side effects [4,5]. Over recent decades, antimicrobial peptides (AMPs) have become popular among researchers [6]. AMPs are short peptides with lengths varying from 10 to 50 amino acids [7]. They are an essential part of innate immunity, acting to protect the host from a wide range of pathogens, including bacteria, fungi, and viruses [8]. Anticancer peptides (ACPs) are a subset of AMPs with anticancer activity [9]. Before introducing the mechanism of ACPs, it is necessary to understand the differences between healthy cells and cancer cells. Firstly, cancer cells carry negatively charged components on their surface, whereas normal cells are electrically neutral [10]. Secondly, cancer cells usually contain a lower amount of membrane cholesterol, making them more flexible and vulnerable to the attack of lysing agents [11]. Nevertheless, some tumors (such as breast and prostate tumors) have a more rigid membrane than normal cells [12]. Such tumors are less likely to be affected by ACPs. The selectivity of ACPs for cancer cells is due to the increased negativity on the cell surface. When positively charged ACPs interact with the membranes of cancer cells, the cell membrane will be lysed. Studies show that ACPs can inhibit the proliferation and immigration of tumor cells and are less likely to cause drug resistance [9,13]. These characteristics of ACPs make them a promising candidate for cancer treatment. Given the promising application of ACPs, it is crucial to identify novel anticancer peptides. Experimental identification is undoubtedly accurate. However, large-scale laboratory identification is often expensive and requires a significant amount of time-consuming processes. Therefore, people resort to computational methods to accelerate the screening process.

Several sequence-based computational methods have been proposed for ACP identification and prediction. Ref. [14] put forward a computational tool called ACPred, which employs both RF and SVM algorithms in the construction of the model. Published in 2020 as an updated version of AntiCP, AntiCP 2.0 produces better results in predicting anticancer peptides [15]. In the same year, ref. [16] proposed a prediction model of AMP called AMPfun. This model uses the random forest (RF) algorithm to identify AMPs and characterize their functional activities. Similarly, ref. [17] employ the gradient boosting decision tree (GBDT) algorithm to establish classifiers and manage to discover novel AMPs on genomic and transcriptomic data. These studies show that machine learning can effectively predict AMPs and ACPs.

In addition to the machine learning algorithms mentioned above, deep neural network (DNN) methods have also been widely applied in AMP recognition problems and have exhibited remarkable performance. Ref. [18] uses a deep learning algorithm that integrates a convolutional [19] and long-short term memory (LSTM) [20] layer to identify AMP sequence patterns. In 2019, ref. [21] proposed an antimicrobial peptide identification model based on DNN. The model employs the embedding layer and the multi-scale convolutional network. The ability of multi-scale convolutional networks to capture latent features enables the model to outperform the state-of-the-art DNN model [18].

These examples illustrate the ability of DNNs to solve protein function prediction problems. However, DNNs also have certain shortcomings [22]. Firstly, the training of DNNs is often arduous because DNNs have too many hyperparameters. Secondly, a large amount of data is usually required when training DNNs. Sometimes, it is difficult to meet the requirement of data size. Moreover, DNN models cannot adjust their architectures according to the input data, making DNNs more complex than needed. Last but not least, neural networks are known as black-box models with limited interpretability. In order to solve these problems, ref. [22] introduced the deep forest algorithm, which follows a layer-by-layer cascade structure similar to a neural network. The training of deep forest does not rely on backpropagation and gradient adjustment. It also has fewer hyperparameters than DNNs, which guarantees it is easy for scientists who do not have so many computational resources to train their models.

Feature selection is also an essential step in the prediction of protein function. It means transforming the original sequence into numeric vectors. There are many ways to achieve this. Commonly used peptide features include amino acid composition (AAC), dipeptide composition (DPC), atomic composition (ATC), etc. Each of them characterizes different aspects of sequence information. In order to maximize the performance of the proposed model, as much information as possible should be incorporated when extracting features from sequences. Feature Extraction based on Graphical and Statistical features (FEGS) was proposed by [23], which is considered one of the most potent approaches for extracting protein sequence features and has demonstrated state-of-the-art performances in a variety of tasks [23,24]. FEGS makes full use of the physicochemical properties of amino acids and statistical information of protein sequences.

In this study, the FEGS module was used to extract graphical features of amino acid sequences together with the evolutionary information and the binary profile of the sequences for use in the model’s training. This paper discards traditional deep neural networks that require complex tuning of parameters and takes an alternative approach instead, namely deep forest, to build our model in a layer-by-layer cascade structure similar to the deep neural network, which enables our proposed method to be more easily transferred to other tasks of sequence analysis. We compared our approach with several baseline algorithms commonly used in sequence analysis tasks. The experiment results demonstrate that our approach outperformed all the baselines. Moreover, comparative experiment results suggest that our presented framework yielded state-of-the-art performance on both elaborate datasets compared to several existing methods, demonstrating the robustness of our framework and leading ability in identifying ACPs and non-ACPs. In addition, the framework proposed in this study is well explainable, which can assist us in further understanding the crucial peptide features. Finally, we released the datasets and code, which can be found at https://github.com/Martinyao1998/GRDF/ (accessed on 1 January 2023).

## 2. Results and Discussion

### 2.1. Evaluation Metrics

In order to evaluate the performance of our presented model, we used four widely used machine learning performance evaluation metrics, including accuracy, precision, recall, and F1-score. The metrics are defined as follows:

(1) $$\begin{array}{cc} \mathrm{Accuracy} & =\frac{\mathrm{TP}\;+\;\mathrm{TN}}{\mathrm{TP}\;+\;\mathrm{TN}\;+\;\mathrm{FP}\;+\;\mathrm{FN}} \end{array}$$

(2) $$\begin{array}{cc} Precision & =\frac{\mathrm{TP}}{\mathrm{TP}+\mathrm{FP}} \end{array}$$

(3) $$\begin{array}{cc} \mathrm{Recall} & =\frac{\mathrm{TP}}{\mathrm{TP}+\mathrm{FN}} \end{array}$$

(4) $$\begin{array}{cc} F1\text{-}\mathrm{score} & =\frac{2\times \mathrm{Precision}\times \mathrm{Recall}}{\mathrm{Precision}\;+\;\mathrm{Recall}} \end{array}$$

where TP, TN, FP, and FN denote the number of true positives, true negatives, false positives, and false negatives, respectively.

### 2.2. Performance Analysis

**Figure 1:**
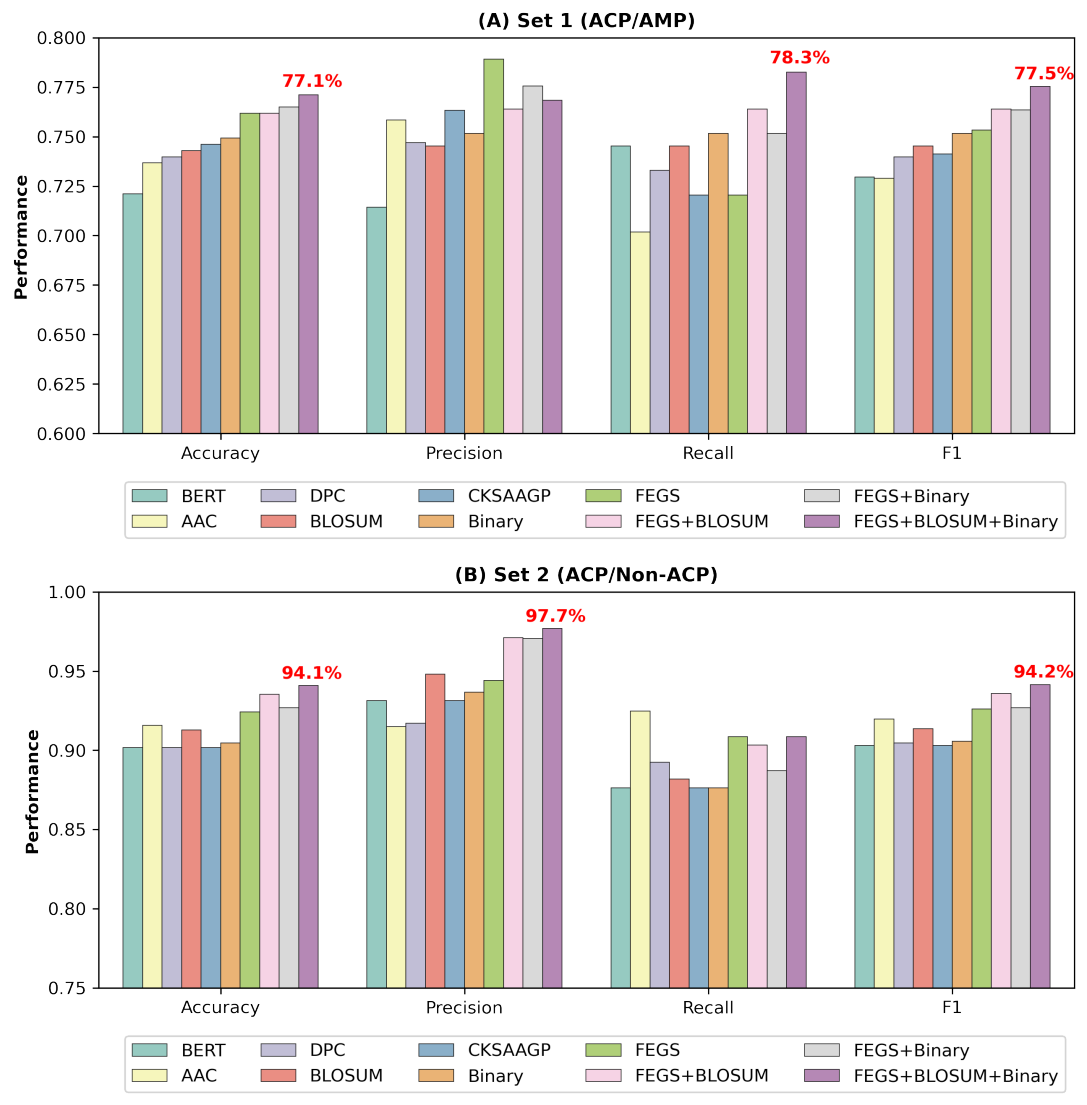
Performance comparison of different features on independent test sets of Set 1 and Set 2.

This paper investigated the contribution of various commonly used sequence features and feature combinations to our deep forest-based model performance. The features investigated here comprised AAC, DPC, CKSAAGP, BLOSUM62, Binary profile, BERT-based features, and FEGS. We conducted experiments on two datasets based on these features, and the results on independent test sets are shown in Figure 1, Supplementary Table S1 (Set 1), and Supplementary Table S2 (Set 2). This study utilized 5-fold cross-validation to optimize the hyperparameters when training the model. The features derived from FEGS reached the best accuracy and F1-score on both datasets compared to AAC, DPC, CKSAAGP, BLOSUM62, Binary Profile, and BERT-based features. Specifically, the features generated by FEGS achieved an accuracy of 76.18% and an F1-score of 75.32% on Set 1 and 92.42% accuracy and 92.60% F1-score on Set 2, demonstrating the strong potential of FEGS for the prediction of ACPs.

In addition to FEGS, the BLOSUM62 matrix and Binary profile also exhibited excellent prediction performance. The BLOSUM62 matrix obtained an F1-score of 74.53% on Set 1 and 91.36% on Set 2. The Binary profile obtained an accuracy of 74.92% and 75.16% F1-score on Set 1, as well as 90.45% accuracy and 90.56% F1-score on Set 2. All this demonstrates the critical importance of the evolutionary information and binary profile of the peptide sequence for the prediction mission of the ACPs.

The experiment further indicates that the combined features of FEGS, BLOSUM62, and Binary profile yielded better results and achieved the best performance on both datasets. More specifically, the combined features achieved an accuracy of 77.12% and an F1-score of 77.54% on Set 1. Meanwhile, an accuracy of 94.1% and an F1-score of 94.15% were attained on Set 2. Figure 1 intuitively depicts the experimental results of the feature comparison, illustrating the superiority of the combined features of FEGS, BLOSUM62, and Binary profile.

In order to derive more reliable results, nested cross-validation experiments were also performed. We utilized five inner folds and five outer folds, and the experiment workflow is shown in Supplementary Figure S1. The reported results were averaged by five outer fold test sets, thus reducing the bias caused by the specificity of individual test sets. The results of the nested cross-validation are summarized in Supplementary Tables S3 (Set 1) and S4 (Set 2). The experimental results of the nested cross-validation further support the previous findings. Specifically, the classifier trained by FEGS achieved the highest mean accuracy and mean F1-score for the classifier trained using a single feature. Moreover, the model trained with the combination of FEGS, BLOSUM62, and Binary profile further boosted prediction performances, achieving the highest mean accuracy and mean F1-scores on both datasets, which is consistent with previous findings.

We compared our model with other ACP prediction classifiers proposed in recent years, including AntiCP [25], AntiCP2 [15], AMPFun [16], dbAMP [17], ACPred [14], iACP-GE [26], StackACPred [27], DeepACP [28], and ACP-MHCNN [29]. The results of the comparison are shown in Table 1. On Set 1, our framework achieved the best accuracy (77.12%), followed by iACP-GE (75.86%) and StackACPred (73.04%). On Set 2, the best accuracy was reached by our framework (94.10%), followed by ACP-MHCNN (92.97%), demonstrating our framework’s superiority in identifying ACPs and non-ACPs.

Furthermore, our framework achieved more balanced prediction results than the others. On Set 1, our presented method achieved the highest precision (76.83%), followed by AntiCP2 (75.78%) and StackACPred (75.51%). As for recall, although our method is lower than several of them, the performances of our method are more balanced. For example, AntiCP achieves 95.65% recall, but its precision is extremely low, at only 52.03%. Similarly, although ACP-MHCNN achieves 94.4% recall, its precision is just 54.28%. This imbalance between precision and recall is not a credible result in classification tasks. On Set 2, our method reached a recall of 97.69%, second only to AntiCP. However, the precision of AntiCP was only 76.88%, which is considered an unbalanced prediction. Similarly, our method achieved a precision of 90.86%, second only to AntiCP2 (91.40%). Nevertheless, our approach is significantly higher on recall (97.69%) than AntiCP2 (92.39%).

As mentioned above, the F1-score is a more unbiased evaluation metric in machine learning tasks. Our framework obtained the highest F1-score on both datasets, reaching 77.54% on Set 1 and 94.15% on Set 2. As a result, compared to the existing methods, our framework could not only accurately discriminate between ACPs and non-ACPs but also achieve a more balanced prediction.

### 2.3. ROC and PR Curves

The Receiver Operating Characteristic (ROC) curves have become a very popular graph-based evaluation criterion for the prediction performance of classification models and have been used in various bioinformatics problems [24,30,31,32]. The ROC curve is a graph indicating the classification model’s performance at all classification thresholds. In addition, the precision–recall (PR) curve is another intuitive graph-based performance evaluation metric which illustrates the trade-off between precision and recall for all thresholds. AUC stands for the area under the curve; more specifically, AUROC denotes the area under the ROC curve, AUPRC denotes the area under the PR curve, and larger areas indicate the model’s better performance.

**Figure 2:**
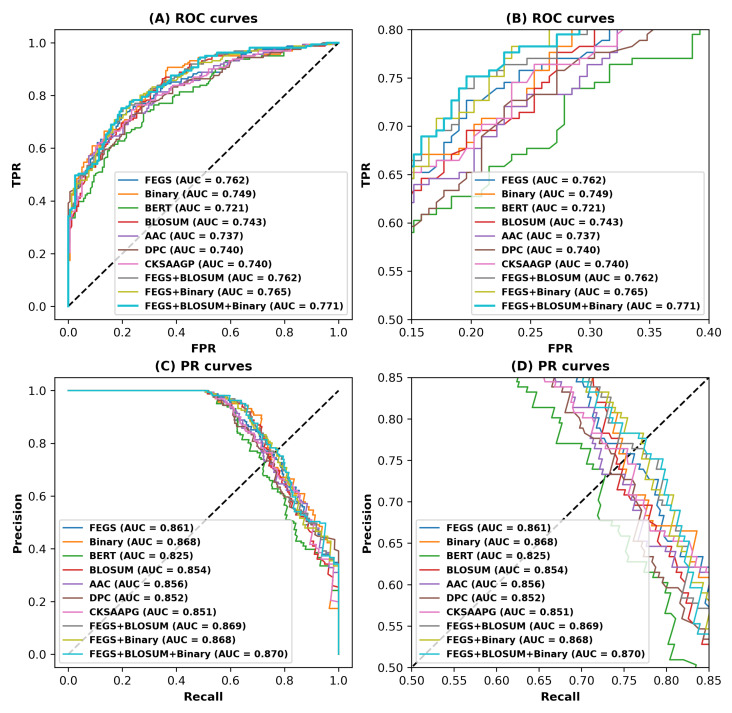
ROC and PR curves for different features or feature combinations on the independent test set of Set 1. (**A**) ROC curves. (**B**) ROC curves for partially enlarged. (**C**) PR curves. (**D**) PR curves for partially enlarged.

In order to more intuitively present the performance of the model, we plotted the ROC and PR curves of the deep forest-based model trained with different features on independent test sets, as shown in Figure 2 and Supplementary Figure S2 for Set 1 and Set 2, respectively. Figure 2A shows the ROC curves for different features on Set 1. Figure 2B shows the same as Figure 2A, but with local enlargements.

It is worth noting that the four curves, which represent the FEGS-based features (FEGS, FEGS+Binary profile, FEGS+BLOSUM, and FEGS+BLOSUM+Binary profile), are distributed in the upper left corner of the ROC curve and wrap around the others, reaching AUROC of 0.762, 0.765, 0.762, and 0.771, respectively, on Set 1, which further confirms the effectiveness of FEGS in the ACP prediction task. Furthermore, the light blue curve represents the combination feature of FEGS, BLOSUM, and Binary profile, which achieves the maximum AUROC (0.771). Similarly, Figure 2C shows the PR curves for the model on Set 1. Figure 2D is the same as Figure 2C, but zoomed in locally. As seen from the figure, the curve representing the features of the combination of FEGS, BLOSUM, and Binary profile achieves the best AUPRC (0.870). A similar conclusion can be drawn from Set 2. As can be seen in Supplementary Figure S2, the model trained using the combined features of FEGS, BLOSUM62, and Binary profile achieves the best AUROC (0.943) and the best AUPRC (0.984). These results discussed above demonstrate the effectiveness of the features used in this study, the superiority of which can be attributed to the following aspects.

Firstly, FEGS fully exploits the physicochemical properties of peptides, incorporating 158 physicochemical properties of amino acids. The physicochemical properties of amino acids are essential for building machine-learning models [33]. Secondly, FEGS employs a novel technique to effectively capture the global information of protein sequences by employing right circular cones where each protein sequence is represented as a 3-dimensional curve. Thirdly, our features incorporate evolutionary information about amino acids. Previous studies suggested that the use of evolutionary information to encode a peptide sequence can be adequate for classification tasks [34,35]. Finally, the binary profile of amino acids is integrated into our features to efficiently encode peptide sequences through a straightforward approach, which is very powerful for predicting different features in multi-omics datasets [36,37,38].

### 2.4. Effectiveness Analysis of the Deep Forest Approach

To further explore the effectiveness of deep forest in this study, we conducted a comparison with other machine learning algorithms on two datasets. Previous work indicated that SVM, RF, and XGBoost achieve relatively better performance than other machine learning methods in the task of AMP prediction [39]. The results compared with other baseline methods on the independent test set are summarized in Table 2. The experiments indicate that the deep forest-based framework outperforms the SVM, random forest, and XGBoost in terms of accuracy, recall, and F1-score on both datasets. The second-ranked method was the random forest. Our deep forest-based approach yielded about two percentage points higher in accuracy and F1-score than the random forest.

Nested cross-validation experiments were also performed to derive more reliable performance comparisons. The results of nested cross-validation are tabulated in Supplementary Table S5. Our proposed framework exhibited the best mean accuracy and F1-score, followed by random forest, which is consistent with previous findings.

Supplementary Figure S3A,D provide a more intuitive view of the performance comparison with other baselines on both datasets. In addition, Supplementary Figure S3B,C,E,F depict their ROC and PR curves, from which it can be seen that the curves in blue representing the deep forest reach larger AUROC and AUPRC compared to other baselines. These results discussed above suggest that the model constructed based on deep forest can more accurately and effectively identify ACPs and non-ACPs. The outstanding performance of the model can be attributed to two reasons.

Firstly, the deep forest-based framework adopts a layer-by-layer cascade architecture for processing features like a DNN. Each level employs random forests and completely random forests for handling features, which are then passed on to the next level. Secondly, the deep forest-based framework employs a strategy to prevent overfitting. When training the model, the number of cascade levels is automatically determined. After each extension of a level, the performance of the current level is estimated. If there is no significant improvement in performance, training will be terminated. Therefore, it is not surprising that our framework achieves the best performance in the classification task of the ACPs.

In addition to the model’s performance, we also need to consider the scope of the audience. Compared with deep learning methods, our framework does not require extensive parameter tuning, which is friendly to scientists with limited computational resources. Hence, it can be stated that our framework is the most powerful prediction method currently available for ACPs. It is both robust in terms of prediction performance and user-friendly for those who have a limited amount of computing resources.

### 2.5. Feature Analysis

**Figure 3:**
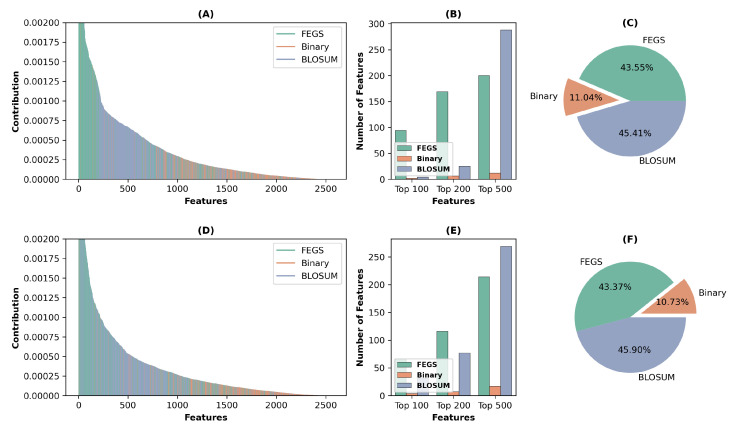
Feature analysis on two datasets. (**A**,**D**) Visualize the three types of features on Set 1 and Set 2 in descending order of their contribution. Green, orange, and blue indicate features in FEGS, Binary profile, and BLOSUM62, respectively. (**B**,**E**) Illustrate the numbers of the top 100, 200, and 500 features on Set 1 and Set 2. (**C**,**F**) Show the proportion of the total contribution of the three types of features.

The splitting rule for random forests is to maximize the reduction in impurity due to splitting [40]. In the case of classification problems, the impurity decrease is usually measured by the Gini index. In general, splits where the impurity is heavily reduced are considered to be critical, and therefore the variables that are used for splits at critical splits are also considered to be significant. Hence, the Gini importance of a variable refers to the mean of the overall reduction in node impurity [41]. This study employs Gini importance to analyze the features we used, and the results are shown in Figure 3.

Figure 3A,D show the features in descending order of their contribution on two datasets, where green, orange, and blue denote features in the FEGS, Binary profile and BLOSUM62, respectively. The vast majority of the features ranked highest for their contribution belongs to FEGS. The number of features in the top 100, top 200, and top 500 are attributable to FEGS, BLOSUM62, and Binary profile, respectively, are plotted in Figure 3B,E. As seen from the figures, on both Set 1 and Set 2, for the top 100 and top 200 features by contribution, the most significant number attributed to FEGS, followed by BLOSUM62, which provides further evidence of the effectiveness of the extraction of our FEGS-based graphical features. Figure 3C,F show the total contribution of the three types of features to the ACP prediction in the two datasets. It can be seen that the two features with the most considerable contribution are BLOSUM62 and FEGS, while the Binary profile makes a relatively small contribution.

## 3. Materials and Methods

In this section, the datasets used in this study are first introduced, followed by the feature extraction module. Next, we elaborate on the architecture of the deep forest.

### 3.1. Dataset Preparation

We employed the benchmark dataset from previous works [15,42] for constructing models in this study. As the length of an ACP is usually between 10 and 50, only peptide sequences with 11 ≤ length ≤ 50 were retained. Finally, two datasets were constructed, the first (Set 1) containing 793 experimentally validated ACPs and 799 negative samples. Notably, the negative samples here are AMPs but do not hold anticancer activities. The second dataset (Set 2) comprises 902 ACPs and 847 random peptides assumed to be non-ACPs.

The two datasets were further cut into train and test sets in a ratio of 8:2, respectively. The train set was used to fit the model during the training process, and the hyperparameters were optimized using a 5-fold cross-validation technique. The test set was used to evaluate the performance of the final model. The sizes of the two datasets are summarized in Table 3.

**Figure 4:**
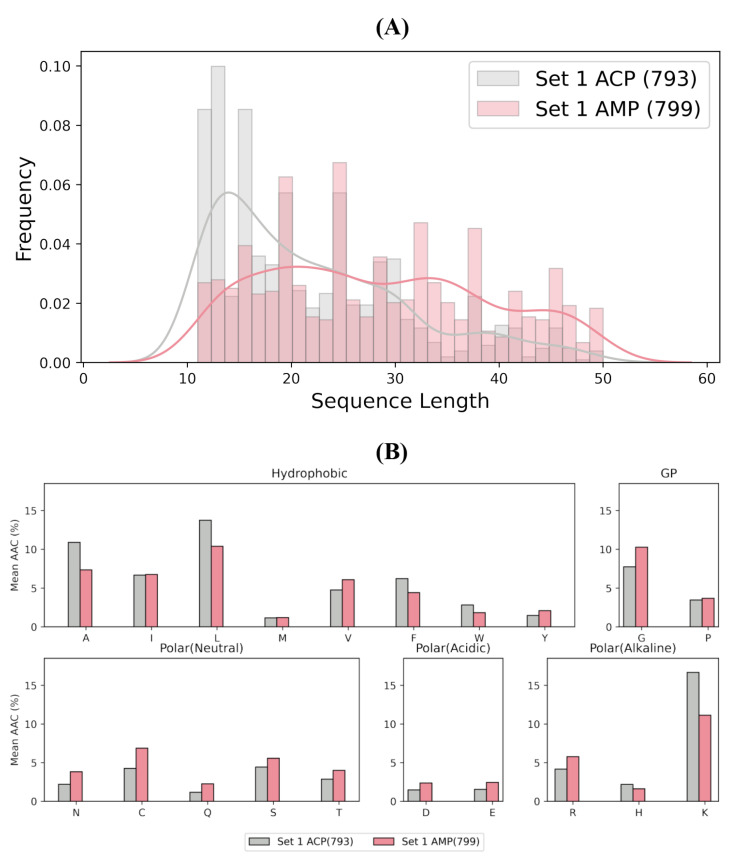
Statistics for Set 1. (**A**) The distribution of amino acid sequence length among positives (ACPs) and negatives (AMPs). (**B**) Mean AAC of positives (ACPs) and negatives (AMPs). The amino acids are grouped according to their physiochemical characteristics.

For better visualization of the difference between ACPs and AMPs, or ACPs and non-ACPs, the distribution of amino acid sequence length among positive and negative sets is shown in Figure 4A and Supplementary Figure S4A, indicating ACPs tend to have shorter amino acid sequences. In addition, we visualize the mean amino acids composition of Set 1 and Set 2, which are given in Figure 4B and Supplementary Figure S4B. Amino acids can be divided into five categories based on their physicochemical properties. Neutral amino acids of ACPs, such as asparagine (N), cysteine (C), glutamine (Q), serine (S), and threonine (T), are less than those of AMPs on Set 1. A similar conclusion can be drawn for the acidic amino acids, including aspartic acid (D) and glutamic acid (E).

### 3.2. Feature Extraction

#### 3.2.1. Graphical Feature Extraction

**Figure 5:**
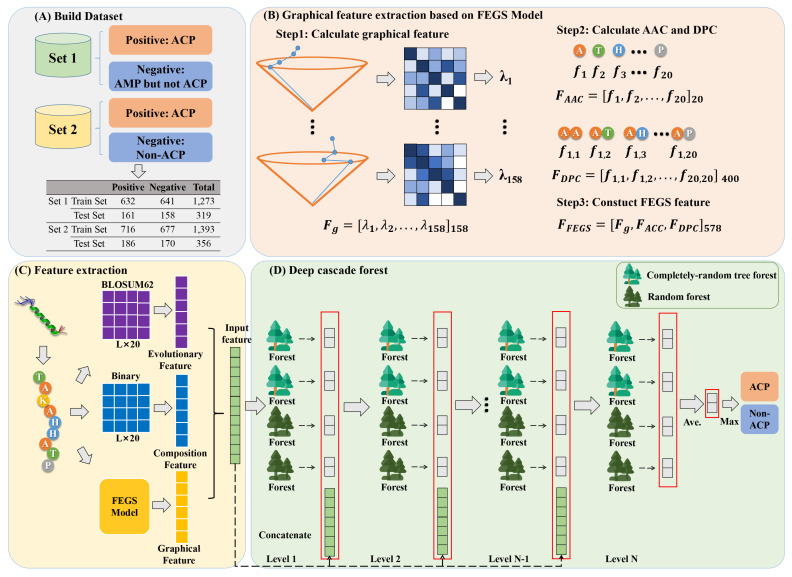
The workflow of GRDF. (**A**) Collection of two datasets (Set 1 and Set 2). (**B**) Graphical feature extraction based on the FEGS model. Each amino acid and amino acid pair is first mapped onto a positive cone of height 1. A 3-dimensional curve is then constructed for each peptide sequence corresponding to each physicochemical property. Each curve is then represented by each symmetry matrix, and this matrix is then characterized by the maximum eigenvalue. In addition, FEGS integrates two statistical features, namely ACC and DPC. (**C**) Feature combination of FEGS, BLOSUM62, and Binary profile. (**D**) Cascade forest architecture. Deep forest performs representation learning of features through a layer-by-layer cascade structure similar to that of deep neural networks. Each layer of the cascade is a forest-based structure.

In this study, we employ FEGS, a feature extraction model of protein sequences, to encode ACP sequences. FEGS was introduced by [23], which efficiently leverages the physicochemical properties of amino acids to encode protein or peptide sequences through a graph-based approach. As illustrated in Figure 5B, each ACP sequence is encoded as a 578-dimensional feature vector by adopting FEGS.

The first step in constructing features using FEGS is to generate their 3D graphical curves for each sequence based on their physicochemical properties. These properties were derived from AAindex, which is a database covering a wide range of physicochemical and biochemical properties of amino acids and amino acid pairs. FEGS has selected 158 indices, each of which contains 20 numerical values representing different properties of 20 different amino acids.

In order to build graphical curves, we first sorted 20 amino acids in ascending order according to their physicochemical indices. Then, 20 amino acids are arranged in order on the curved face of a right cone with a height of 1. The position of each amino acid can be represented by the following equation:

(5) $$\begin{array}{cc} \phi (\Omega_{i}) & =\left(\cos\frac{2\pi i}{20},\sin\frac{2\pi i}{20},1\right),i=1,2,\ldots ,20 \end{array}$$

where $\Omega_{i}$ denotes each of the 20 amino acids. The 400 amino acid pairs are subsequently mapped to the underside of the cone in accordance with the following equation:

(6) $$\begin{array}{cc} \psi (\Omega_{i}\Omega_{j}) & =\phi \left(\Omega_{i}\right)+\frac{1}{4}\left(\phi \left(\Omega_{j}\right)-\phi \left(\Omega_{i}\right)\right),i,j=1,2,\ldots ,20 \end{array}$$

where $\Omega_{i}\Omega_{j}$ corresponds to each of the 400 amino acid pairs.

Based on this feature representation method, a three-dimensional spatial curve can be constructed for each physicochemical property of each ACP. Given a sequence *S* with *N* amino acids $S=s_{1}s_{2}\ldots s_{N}$, the curve it corresponds to can be constructed by extending a 3D path in a right cone as follows. Starting from the origin $P_{0}\left(0,0,0\right)$, the curve extends to the point $P_{1}=\left(x_{1},y_{1},z_{1}\right)$, then to $P_{2}=\left(x_{2},y_{2},z_{2}\right)$ until $P_{N}=\left(x_{N},y_{N},z_{N}\right)$, where $P_{1},P_{2},\ldots ,P_{N}$ correspond to amino acids $s_{1},s_{2},\ldots ,s_{N}$, respectively. The coordinate of point $P_{i}$ is determined by the following equation.

(7) $$\begin{array}{cc} \psi (S_{i}) & =\psi \left(S_{i-1}\right)+\phi \left(S_{i}\right)+\sum_{\Omega_{1},\Omega_{2}\in A,C,D,\ldots Y}f_{\Omega_{1}\Omega_{2}}\cdot \phi \left(\Omega_{1}\Omega_{2}\right) \end{array}$$

Here, $\psi \left(S_{0}\right)=\left(0,0,0\right)$ and $f_{\Omega_{1}\Omega_{2}}$ are calculated by the frequency of the amino acid pair $\Omega_{1}\Omega_{2}$ in the subsequence of the first *i* amino acids of the sequence *P*. By this means, 158 unique spatial curves are constructed for each sequence, with each curve corresponding to a property.

Next, a non-negative symmetric matrix *M* is utilized to represent each constructed curve. The off-diagonal entry $M_{ij}\left(i\neq j\right)$ is defined as a quotient of the Euclidean distance between $P_{i}$ and $P_{j}$ and the sum of geometrical lengths of the edge between two points along the curve. The diagonal entries of *M* are set to 0. Then, the largest eigenvalue of *M* divided by the sequence length is computed to represent this matrix. This process is repeated in each space curve that represents a property, finally generating a 158-dimensional feature as shown below:

(8) $$\begin{array}{cc} F_{g} & ={\left[\lambda_{1},\lambda_{2},\ldots ,\lambda_{158}\right]}_{158} \end{array}$$

In addition, two statistical features, amino acid composition (AAC), and dipeptide composition (DPC), are also incorporated in this feature extraction model. AAC is defined as the frequency of an amino acid in a sequence, which can be calculated as follows:

(9) $$\begin{array}{cc} F_{ACC} & ={\left[f_{1},f_{2},\ldots ,f_{20}\right]}_{20} \end{array}$$

where $f_{i}$ is the frequency of the *i*-th amino acid. By this expression, $F_{ACC}$ is a 20-dimensional vector with the sum of all dimensions equal to 1.

In a similar way to AAC, DPC is defined as the frequency of occurrence of an amino acid pair, which gives a calculation as follows:

(10) $$\begin{array}{cc} F_{DPC} & ={\left[f_{1,1},\ldots ,f_{i,j},\ldots ,f_{20,20}\right]}_{400} \end{array}$$

where $f_{i,j}$ denotes the frequency of occurrence of an amino acid pair *i*-th and *j*-th. A total of 400(=20×20) combinations of amino acid pairs are available, so $F_{DPC}$ is a 400-dimensional vector, and the sum of all dimensions is equal to 1.

Finally, the feature generated by FEGS can be constructed by concatenating $F_{g},F_{ACC}$, and $F_{DPC}$, which contains 578(=158+20+400) dimensions and has the following form.

(11) $$\begin{array}{cc} F_{FEGS} & ={\left[F_{g},F_{ACC},F_{DPC}\right]}_{578} \end{array}$$

#### 3.2.2. Evolutionary Information Representation

The evolutionary information of proteins plays a significant factor in the task of protein analysis [24,32,35]. In order to efficiently extract evolutionary information from peptides, we adopt the BLOSUM62 scoring matrix to encode ACP sequences. The BLOSUM matrix was first introduced by [43], which is used to score sequence alignments that are evolutionarily divergent. Several sets of BLOSUM matrices, named with numbers, are designed to compare sequences with different evolutionary distances. Matrices with large numbers closest are used in evolutionarily close sequences. BLOSUM62 matrix is built using sequences with less than 62% similarity. It is also the default matrix for protein Basic Local Alignment Search Tool (BLAST). A peptide with length *L* can be represented by an L×20 matrix, as shown in the following:

(12) $$\begin{array}{cc} \mathrm{BLOSUM}62 & =\left[\begin{array}{cccc} p_{1,1} & p_{1,2} & \ldots & p_{1,20}\\ p_{2,1} & p_{2,2} & \ldots & p_{2,20}\\ \vdots & \vdots & \ddots & \vdots \\ p_{L,1} & p_{L,2} & \ldots & p_{L,20} \end{array}\right] \end{array}$$

where $p_{n,i}$ represents the similarity between the n−th amino acid in the peptide and one of the 20 amino acids. Integrating evolutionary information enables our model to gain a deeper understanding of the similarities between sequences.

#### 3.2.3. Binary Profile Representation

The binary profile is a critical feature in constructing sequence-based models and has been utilized in multiple prediction tasks [29,44,45]. This study adopts the binary profile to encode the amino acid sequence, which reflects the composition and order information of 20 amino acids in a protein sequence by uniquely encrypting each amino acid based on the one-hot encoding method. It converts each amino acid into a 20-dimensional binary vector. One example is Ala which can be represented as a vector [1,0,0,0,0,0,0,0,0,0,0,0,0,0,0,0,0,0,0,0]. Thus, a peptide of length *L* can be represented as an L×20 dimensional matrix.

Due to the different lengths of ACPs, BLOSUM62 and Binary profile are converted to a zero-padded numeric matrix of size 50×20 to fit the datasets. The final features are composed of FEGS, BLOSUM62, and Binary profile, which are used as input to the deep forest module.

### 3.3. Deep Forest Architecture

The deep forest is an ensemble learning model that has been proposed in the last two years and is considered an alternative to deep learning. The success of deep learning relies on its remarkable capacity to characterize original features. Similarly, the deep forest employs a tree-based cascade structure, which discards the traditional derivable neurons and replaces them with decision trees to capture higher-level features. This cascade structure is shown in Figure 5D, where each level of the cascade receives the features from the previous cascade, conducts further feature processing to obtain better-characterized features, and then passes to the next level of the cascade.

**Figure 6:**
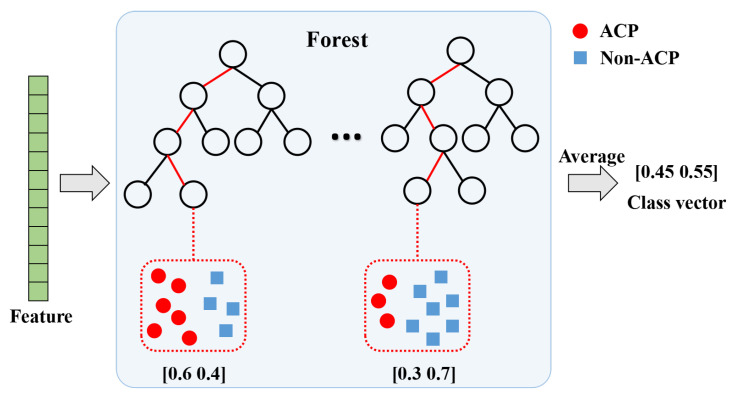
The flowchart for generation of category vectors. Different markers on the leaf nodes indicate various categories. The prediction of ACP is a binary classification mission; therefore, there are two types of markers on the leaf nodes, where red and blue dots represent ACPs and non-ACPs, respectively.

It is well known that diversity is one of the most critical factors affecting the performance of the ensemble model. To increase the diversity, each level of the cascade comprises random forests and completely random forests. Thus, each layer can be viewed as an ensemble of ensembles. Each forest processes the input information and then generates the class vector, which is an estimate of class distribution. The procedure for generating class vectors for each forest is illustrated in Figure 6. Firstly, each tree first calculates the percentage of samples belonging to different categories that fall on the leaf nodes. The average is then taken over each category distribution on all trees in the same forest. Our mission is a binary classification task, i.e., to predict whether given peptides are ACP or not. As illustrated in Figure 6, the red segment is the path of the instance into the leaf node of each tree, and red and blue dots denote ACPs and non-ACPs, respectively. Therefore, the final output of each forest is a 2-dimensional vector. Assuming that there are *n* random forests and n completely random forests in each layer, concatenating their generated class vectors together yields a 4n-dimensional augmented feature. Let us suppose that the initial features are *k*-dimensional, and the input features of each layer are of dimension k+4n, except for the first layer. Here, the number of random forests is a hyperparameter, which is determined by cross-validation during the training process. After the last layer generates the augmented feature vector, each dimension of all the augmented vectors is averaged to obtain a 2-dimensional vector, and each dimension of this 2-dimensional vector indicates ACP or non-ACP. The label corresponding to the maximum dimension of this vector is used as the result of the prediction.

## 4. Conclusions

Cancer is one of the most severe diseases worldwide, and there is no perfectly effective treatment to date. Peptide-based therapies are drawing increasing attention due to their high specificity and low side effects. Hence, the identification of ACPs is significant in facilitating peptide-based therapeutic approaches to healing cancer. The development of computer-aided drugs provides an opportunity to discover novel ACPs. In this study, we employ a novel technique to represent each peptide sequence as 3D spatial curves with the aid of right cones in order to obtain more efficient global descriptions. The excellent performance of deep learning is due to its layer-by-layer cascade architecture. However, the sheer volume of data it requires and the complexity of hyperparameter tuning are prohibitive to many people, especially researchers with limited computational resources. Therefore, this study proposes an alternative to deep learning, namely deep forest, to identify ACPs. It employs a similar cascade architecture with powerful feature representation capabilities, not requiring complex hyperparameter tuning. Therefore, it is straightforward to transfer to other tasks of bio-sequence analysis. Our approach demonstrates stronger robustness on both datasets than algorithms commonly used in sequence analysis tasks. The framework in this study outperforms currently existing ACP prediction methods and achieves state-of-the-art performance, which demonstrates the strong potential of our framework in recognizing anticancer peptides. In addition, our model is well interpretable, which is beneficial in aiding scientists to better understand the sequence features of peptides. We are confident that this study could aid researchers in facilitating the discovery of anticancer peptides and contribute to the development of novel cancer therapies.

## Figures and Tables

**Table 1:** Performance comparison with the other ACP prediction models proposed in recent years on independent test sets. Best performance values are in bold.

Dataset	Method	Accuracy	Precision	Recall	F1
Set 1	AntiCP	53.29%	52.03%	**95.65%**	67.40%
	AntiCP2	70.22%	75.78%	62.25%	67.13%
	AMPFun	68.65%	67.84%	72.05%	69.88%
	dbAMP	67.40%	63.77%	81.99%	71.74%
	ACPred	54.86%	53.23%	86.96%	66.04%
	iACP-GE	75.86%	75.30%	77.63%	76.45%
	StackACPred	73.04%	75.51%	68.94%	72.07%
	DeepACP	57.99%	55.05%	91.30%	68.69%
	ACP-MHCNN	57.05%	54.28%	94.40%	68.93%
	**This study**	**77.12%**	**76.83%**	78.26%	**77.54%**
Set 2	AntiCP	87.92%	**100.00%**	76.88%	86.93%
	AntiCP2	91.57%	92.39%	**91.40%**	91.89%
	AMPFun	77.25%	89.47%	63.98%	74.61%
	dbAMP	49.72%	51.71%	56.99%	54.22%
	ACPred	88.48%	89.62%	88.17%	88.89%
	iACP-GE	89.60%	92.57%	87.09%	89.75%
	StackACPred	92.97%	96.00%	90.32%	93.07%
	DeepACP	90.73%	93.60%	87.97%	90.70%
	ACP-MHCNN	91.57%	95.34%	88.17%	91.62%
	**This study**	**94.10%**	97.69%	90.86%	**94.15%**

**Table 2:** Performance comparison results with baseline methods on independent test sets. Best performance values are in bold.

	Method	Accuracy	Precision	Recall	F1
Set 1	SVM	74.29%	**78.42%**	67.70%	72.67%
	XGBoost	73.04%	72.73%	74.53%	73.62%
	RF	75.24%	75.95%	74.53%	75.24%
	**This study (Deep forest)**	**77.12%**	76.83%	**78.26%**	**77.54%**
Set 2	SVM	87.36%	**97.96%**	77.42%	86.49%
	XGBoost	92.13%	94.89%	89.78%	92.27%
	RF	92.70%	97.62%	88.17%	92.66%
	**This study (Deep forest)**	**94.10%**	97.69%	**90.86%**	**94.15%**

**Table 3:** Overview of two datasets.

		Positive	Negative	Total	Description of Positives and Negatives
Set 1	Train Set	632	641	1273	The positives were anticancer peptides. The
	Test Set	161	158	319	negatives were antimicrobial peptides other
					than anticancer peptides.
Set 2	Train Set	716	677	1393	The positives were anticancer peptides. The
	Test Set	186	170	356	negatives were chosen at random from the
					Swiss-Prot database.

## Data Availability

GRDF and datasets of this study are available at https://github.com/Martinyao1998/GRDF/ (accessed on 1 January 2023).

## Supplementary Materials

The following supporting information can be downloaded at: https://www.mdpi.com/article/10.3390/ijms24054328/s1.
