# Supplementary material for: Accelerating the Discovery of Anticancer Peptides through Deep Forest Architecture with Deep Graphical Representation
Source: Int J Mol Sci. 2023 Feb 21;24(5):4328. doi: 10.3390/ijms24054328 (PMC10001941; doi:10.3390/ijms24054328)
Supplement: Supplementary file 1 [file ijms-24-04328-s001.zip › ijms-2178631-supplementary.pdf]

SUPPLEMENTARY MATERIALS

Table S1. Performance comparison of models built with different features on the independent test set of Set 1.

Table S2. Performance comparison of models built with different features on the independent test set of Set 2.

Table S3. Performance summary of nested cross-validation on Set 1. The reported results are taken by the [mean $\pm$ Std] value of test sets within 5 outer folds.

Table S4. Performance summary of nested cross-validation on Set 2. The reported results are taken by the [mean $\pm$ Std] value of test sets within 5 outer folds.

Table S5. Performance comparison with baseline methods (nested cross-validation). The reported results are taken by the [mean $\pm$ Std] value of test sets within 5 outer folds.

Figure S1. Diagram of nested cross-validation. We adopted five outer folds and five inner folds. The experiment results were derived by averaging the test sets of the five outer folds.

Figure S2. ROC and PR curves for different features or feature combinations on the independent test set of Set 2. (A) ROC curves. (B) ROC curves for partially enlarged. (C) PR curves. (D) PR curves for partially enlarged.

Figure S3. Performance comparison with baseline methods on independent test sets. (A) and (D) show comparisons in terms of Accuracy, Precision, Recall, and F1-score on Set 1 and Set 2, respectively. (B) and (E) plot the ROC curves of the deep forest-based model and baselines on Set 1 and Set 2, respectively. (C) and (F) plot the PR curves of the deep forest-based model and baselines on Set 1 and Set 2, respectively.

Figure S4. Statistics for Set 2. (A) The distribution of amino acid sequence length among positives (ACPs) and negatives (non-ACPs). (B) Mean AAC of positives (ACPs) and negatives (non-ACPs). The amino acids are grouped according to their physiochemical characteristics.

Table S1. Performance comparison of models built with different features on the independent test set of Set 1.

Feature	Accuracy	Precision	Recall	F1
BERT	72.10%	71.43%	74.53%	72.95%
AAC	73.67%	75.84%	70.19%	72.90%
DPC	73.98%	74.68%	73.29%	73.98%
BLOSUM	74.29%	74.53%	74.53%	74.53%
CKSAAGP	74.61%	76.32%	72.05%	74.12%
Binary	74.92%	75.16%	75.16%	75.16%
FEGS	76.18%	**78.91%**	72.05%	75.32%
FEGS+BLOSUM	76.18%	76.40%	76.40%	76.40%
FEGS+Binary	76.49%	77.56%	75.16%	76.34%
FEGS+BLOSUM+Binary	**77.12%**	76.83%	**78.26%**	**77.54%**

Table S2. Performance comparison of models built with different features on the independent test set of Set 2.

Feature	Accuracy	Precision	Recall	F1
BERT	90.17%	93.14%	87.63%	90.30%
AAC	91.57%	91.49%	**92.47%**	91.98%
DPC	90.17%	91.71%	89.25%	90.46%
BLOSUM	91.29%	94.80%	88.17%	91.36%
CKSAAPG	90.17%	93.14%	87.63%	90.30%
Binary	90.45%	93.68%	87.63%	90.56%
FEGS	92.42%	94.41%	90.86%	92.60%
FEGS+BLOSUM	93.54%	97.11%	90.32%	93.59%
FEGS+Binary	92.70%	97.06%	88.71%	92.70%
FEGS+BLOSUM+Binary	**94.10%**	**97.69%**	90.86%	**94.15%**

Table S3. Performance summary of nested cross-validation on Set 1. The reported results are taken by the [mean ± Std] value of test sets within 5 outer folds.

**Experiment**	**Feature (Method)**	**Accuracy**	**Precision**	**Recall**	**F1**
Feature comparison	CKSAAGP	73.99%±0.01	79.47%±0.06	64.80%±0.04	71.19%±0.02
	AAC	75.25%±0.02	79.28%±0.02	68.16%±0.05	73.20%±0.03
	BERT	74.69%±0.02	77.23%±0.03	69.98%±0.04	73.34%±0.02
	DPC	75.82%±0.02	80.43%±0.02	68.08%±0.07	73.56%±0.04
	Binary	75.32%±0.02	77.92%±0.02	70.38%±0.06	73.86%±0.03
	BLOSUM	75.25%±0.03	77.13%±0.03	71.34%±0.06	74.07%±0.04
	FEGS	76.76%±0.02	**82.30%±0.04**	67.91%±0.05	74.35%±0.04
	FEGS+BLOSUM	76.44%±0.02	79.83%±0.04	70.78%±0.05	74.88%±0.03
	FEGS+Binary	77.20%±0.02	80.83%±0.03	71.31%±0.05	75.64%±0.03
	FEGS+BLOSUM+Binary	**77.95%±0.03**	81.33%±0.04	**72.59%±0.02**	**76.64%±0.02**

Table S4. Performance summary of nested cross-validation on Set 2. The reported results are taken by the [mean ± Std] value of test sets within 5 outer folds.

**Experiment**	**Feature (Method)**	**Accuracy**	**Precision**	**Recall**	**F1**
Feature comparison	CKSAAGP	89.02%±0.01	90.82%±0.04	87.59%±0.01	89.13%±0.02
	DPC	90.62%±0.01	92.40%±0.03	89.20%±0.01	90.73%±0.01
	Binary	90.91%±0.02	94.71%±0.05	87.30%±0.02	90.78%±0.02
	BERT	91.08%±0.02	93.06%±0.03	89.37%±0.03	91.14%±0.02
	BLOSUM	91.54%±0.01	95.75%±0.01	87.44%±0.02	91.39%±0.01
	AAC	91.65%±0.02	93.09%±0.02	**90.42%±0.03**	91.70%±0.02
	FEGS	91.94%±0.01	95.22%±0.01	88.77%±0.02	91.88%±0.01
	FEGS+BLOSUM	92.28%±0.02	**95.98%±0.02**	88.82%±0.02	92.24%±0.02
	FEGS+Binary	92.22%±0.01	95.12%±0.02	89.58%±0.01	92.25%±0.01
	FEGS+BLOSUM+Binary	**92.85%±0.01**	**95.98%±0.01**	89.85%±0.02	**92.81%±0.01**

Table S5. Performance comparison with baseline methods (nested cross-validation). The reported results are taken by the [mean ± Std] value of test sets within 5 outer folds.

Dataset	Method	Accuracy	Precision	Recall	F1
Set 1	SVM	74.56%±0.02	**81.87%±0.05**	62.86%±0.02	71.07%±0.03
	XGBoost	74.68%±0.04	76.22%±0.04	71.51%±0.04	73.77%±0.04
	Random forest	75.75%±0.04	79.15%±0.03	69.42%±0.07	73.90%±0.05
	This study (Deep forest)	**77.95%±0.03**	81.33%±0.04	**72.59%±0.02**	**76.64%±0.02**
Set 2	SVM	87.19%±0.02	**98.17%±0.01**	76.68%±0.04	86.07%±0.02
	XGBoost	91.20%±0.01	92.99%±0.01	89.65%±0.02	91.28%±0.02
	Random forest	91.59%±0.01	96.17%±0.02	89.10%±0.01	91.40%±0.01
	This study (Deep forest)	**92.85%±0.01**	95.98%±0.01	**89.85%±0.02**	**92.81%±0.01**

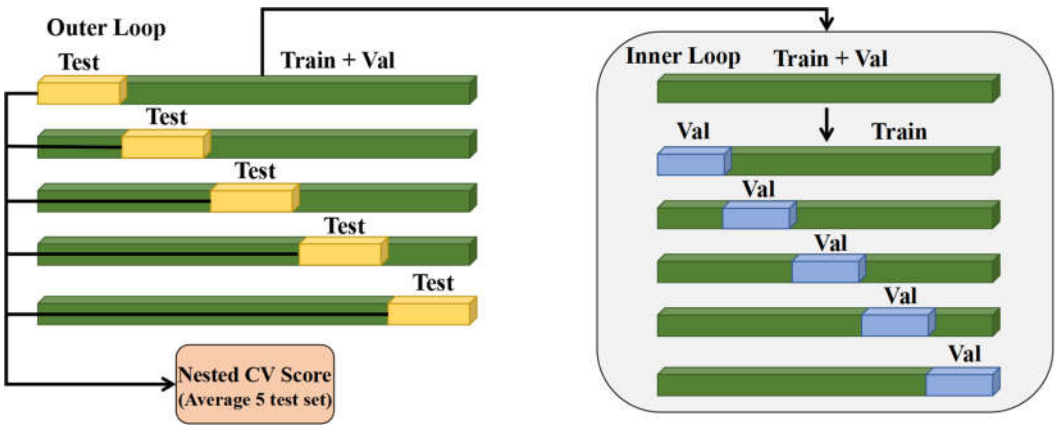

Figure S1. Diagram of nested cross-validation. We adopted five outer folds and five inner folds. The experiment results were derived by averaging the test sets of the five outer folds.

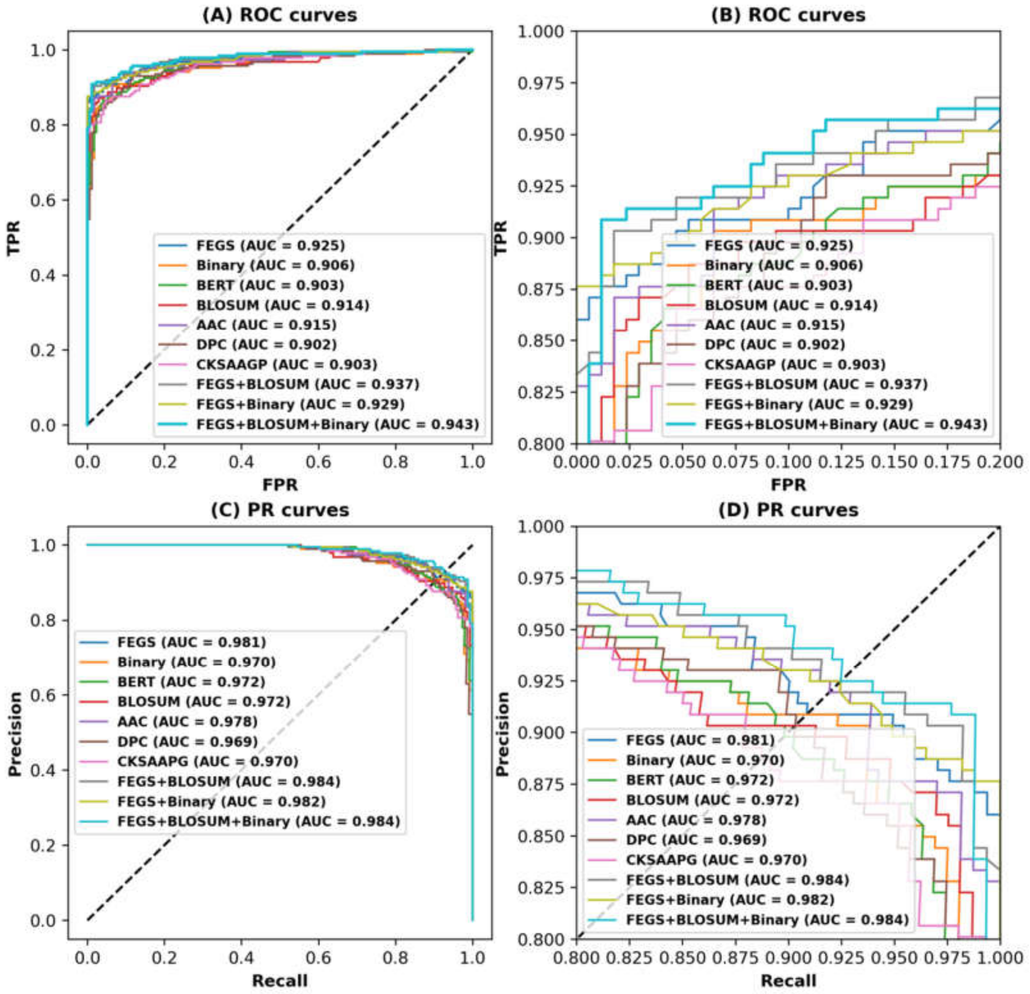

Figure S2. ROC and PR curves for different features or feature combinations on the independent test set of Set 2. (A) ROC curves. (B) ROC curves for partially enlarged. (C) PR curves. (D) PR curves for partially enlarged.

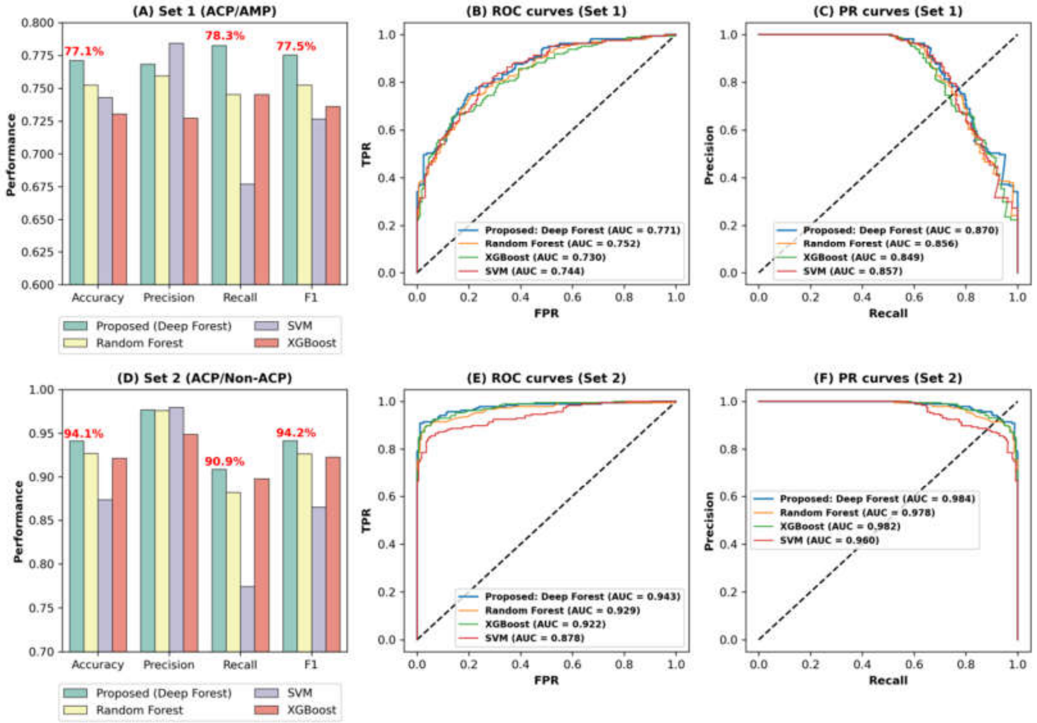

Figure S3. Performance comparison with baseline methods on independent test sets. (A) and (D) show comparisons in terms of Accuracy, Precision, Recall, and F1-score on Set 1 and Set 2, respectively. (B) and (E) plot the ROC curves of the deep forest-based model and baselines on Set 1 and Set 2, respectively. (C) and (F) plot the PR curves of the deep forest-based model and baselines on Set 1 and Set 2, respectively.

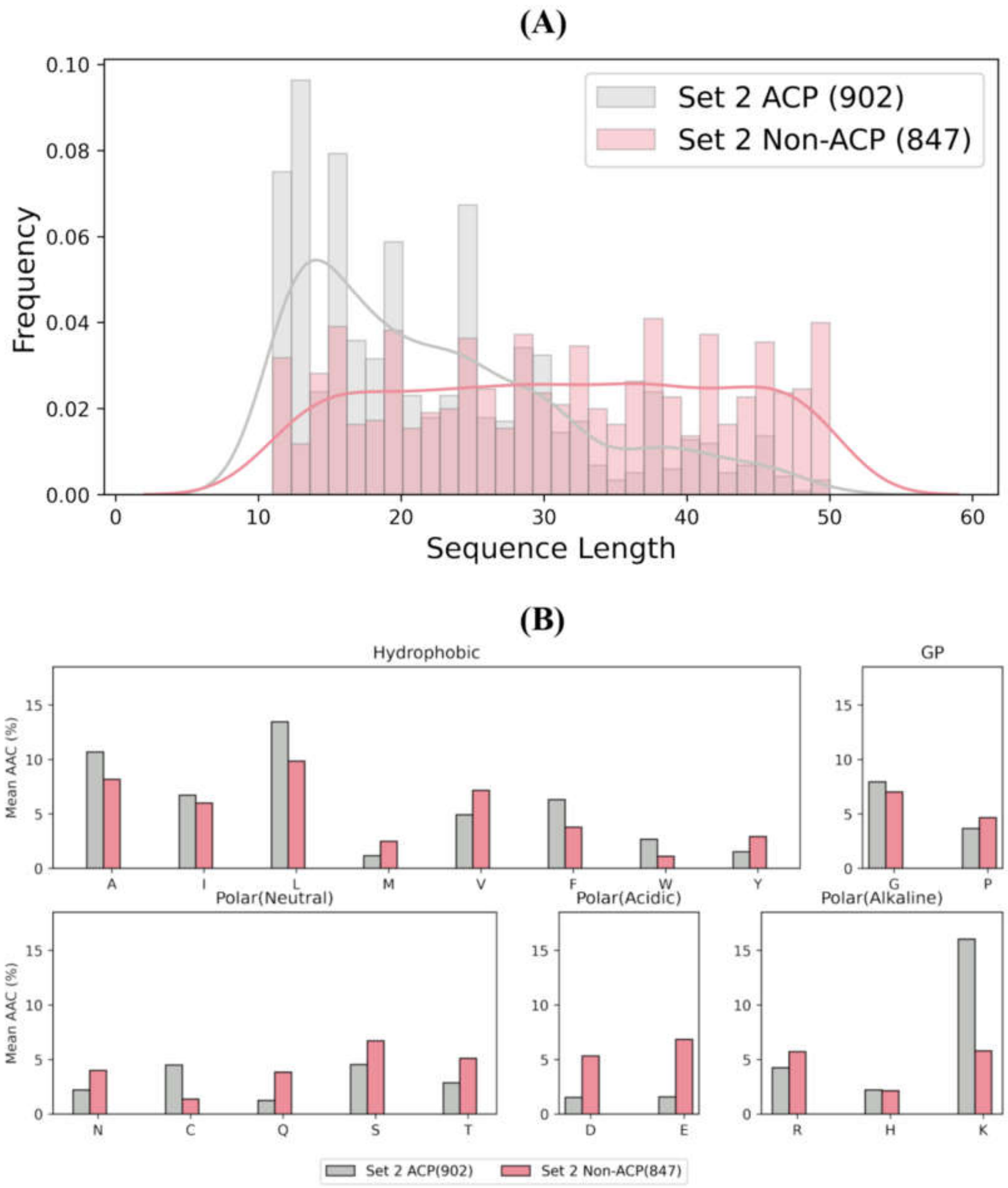

Figure S4. Statistics for Set 2. (A) The distribution of amino acid sequence length among positives (ACPs) and negatives (non-ACPs). (B) Mean AAC of positives (ACPs) and negatives (non-ACPs). The amino acids are grouped according to their physiochemical characteristics.
